# Generalized Peritonitis Secondary to Perforated Uterine Pyometra

**DOI:** 10.7759/cureus.29938

**Published:** 2022-10-05

**Authors:** Jessica Biller, Bradley S Winegardner, Michael Sleet

**Affiliations:** 1 General Surgery, Conemaugh Memorial Medical Center, Johnstown, USA; 2 Surgery, Conemaugh Memorial Medical Center, Johnstown, USA

**Keywords:** pneumouterus, purulent peritonitis, pneumoperitoneum, generalized peritonitis, pyometra, spontaneous uterine rupture

## Abstract

Generalized peritonitis with sepsis is a common general surgery emergency. The most likely implicated structure for generalized peritonitis with pneumoperitoneum is the gastrointestinal tract with urgent explorative laparotomy being the most definitive treatment. In this particular case, perforated diverticulitis was suspected and upon an exploration of the abdomen, frank pus in the setting of normal colon was noted. Some common differential diagnoses for frank pus in the abdomen include viscus perforation, pancreatic necrosis, gangrenous cholecystitis, or penetrating abdominal trauma. Here, we report a rare occurrence of peritonitis secondary to uterine rupture from pyometra.

## Introduction

Pyometra is defined as the accumulation of pus within the uterine cavity secondary to the inability to adequately drain the uterine cavity and accounts for only 0.038% of gynecologic-related hospital admissions [1]. A PubMed data search found less than 50 current case reports on nongravid spontaneous uterine rupture secondary to pyometra. Classically, the presentation is a post-menopausal female with purulent vaginal discharge and pelvic pain [2]. Uterine rupture is secondary to a stenotic cervix in which vaginal discharge is unable to properly drain thus creating a “plug". This forces a build-up of pressure within the uterus and can lead to perforation. Ninety percent of the time, purulent peritonitis is due to gastrointestinal perforation, not a genitourinary source [2,3]. Patients will present with generalized peritonitis and pneumoperitoneum on imaging. 

## Case presentation

**Figure 1:**
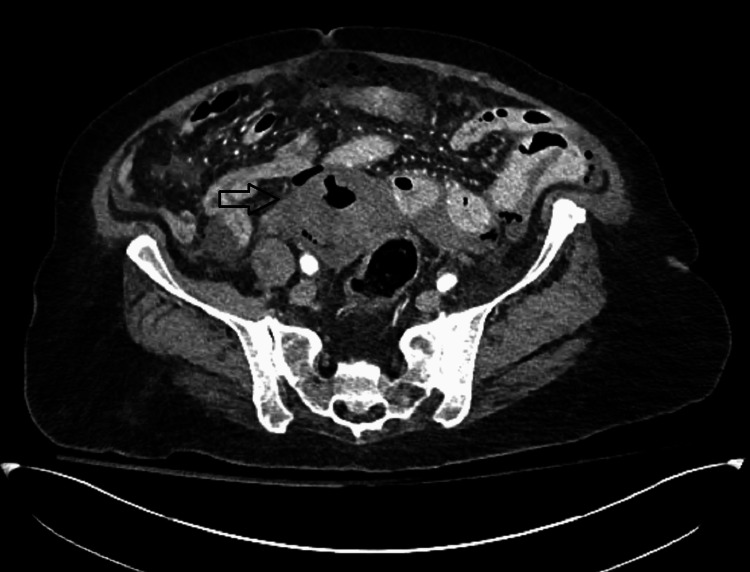
Axial cut from CT abdomen/pelvis showing intraperitoneal and periuterine free air

**Figure 2:**
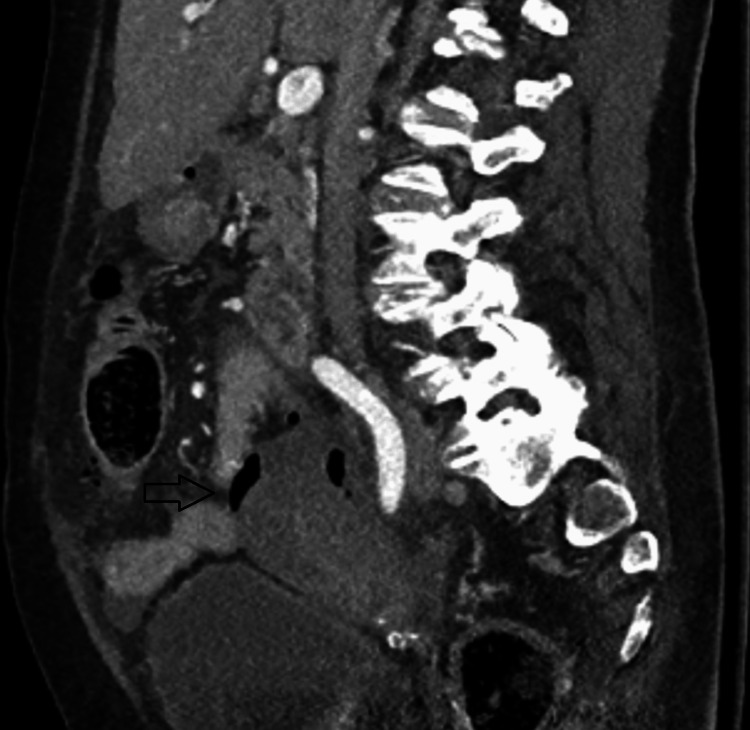
Sagittal cut from CT abdomen/pelvis showing periuterine free air

Our case reports an 80-year-old female with a history of type 2 diabetes mellitus who presented to the emergency department with abdominal pain, vomiting, diarrhea, and fever for one day. Per the family, the patient had also been complaining of generalized weakness and falls in the preceding week. She was hypotensive on presentation, which responded to fluid resuscitation. Her abdominal exam revealed diffuse tenderness to palpation with distention. She had a leukocytosis of 13,000, bandemia of 30%, and lactic acidosis of 4 mmol/L. She underwent computed tomography (CT) of the abdomen and pelvis, which revealed pneumoperitoneum. Figures 1, 2 show the CT findings that reveal periuterine edema, pneumoperitoneum, pneumouterus, and periuterine free air. This was interpreted by radiology to be secondary to perforated sigmoid diverticulitis.

**Figure 3:**
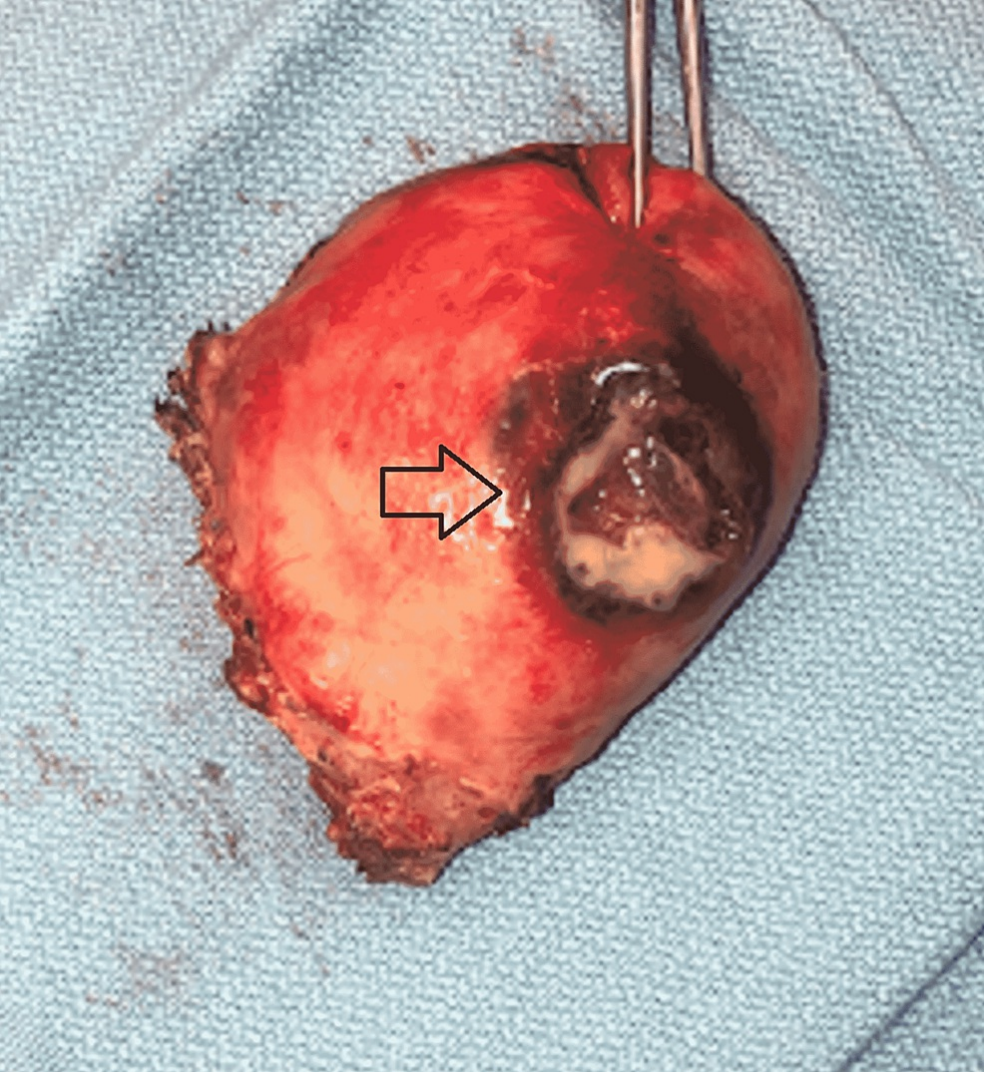
Surgical pathology showing gross uterine perforation

**Figure 4:**
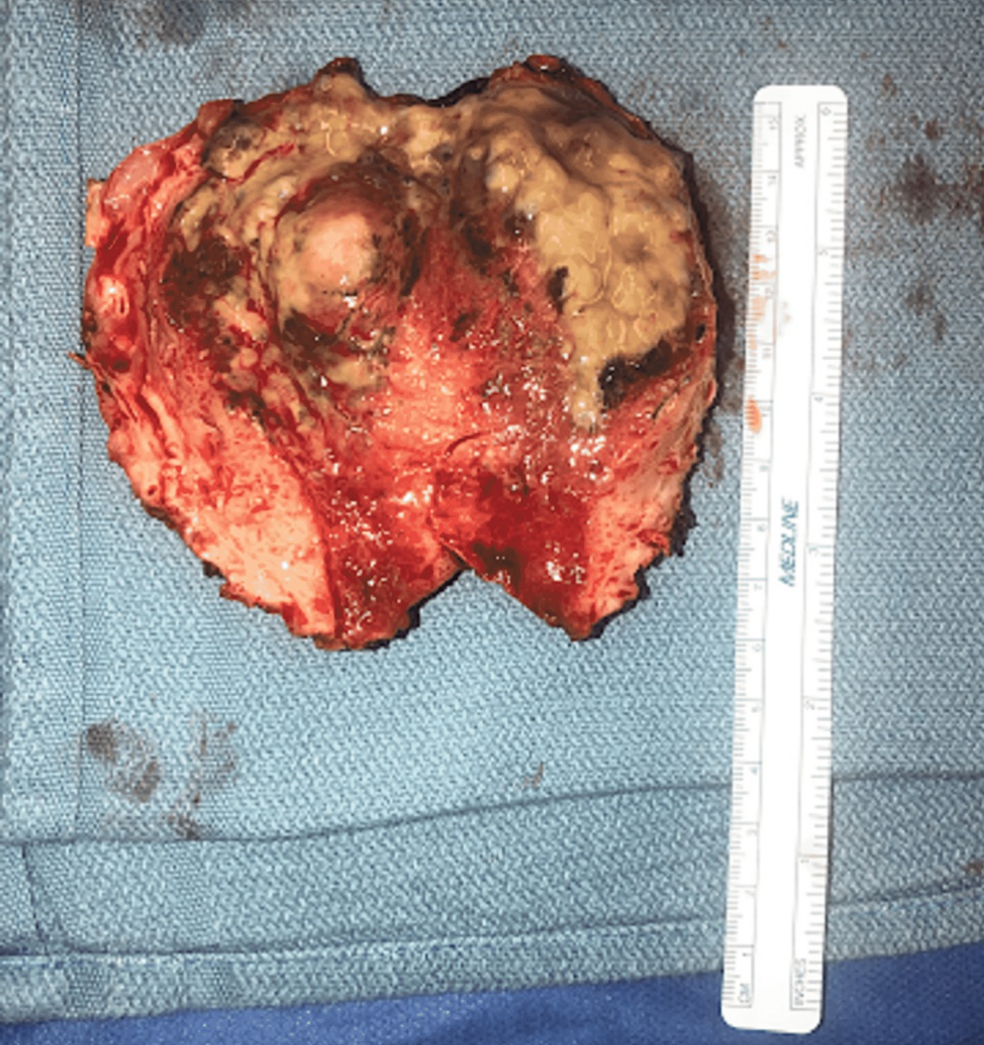
Surgical pathology showing purulent-appearing intrauterine cavity

She was immediately taken to the operating room for an exploratory laparotomy, which revealed purulent peritonitis and normal appearing bowel. Intraoperative cultures were obtained which grew *Bacteroides fragilis* and *Escherichia coli*. A perforation was found at the posterior wall of the uterus, which was leaking frank pus (Figure 3). The uterus along with the adnexa and fallopian tubes appeared hyperemic and dusky; therefore, gynecology was consulted intra-operatively. The patient underwent a hysterectomy with bilateral salpingo-oophorectomy. She was transferred to the surgical intensive care unit (ICU) postoperatively for continued resuscitation and antibiotic therapy. She was increasingly unstable requiring vasopressor support and ultimately expired on postoperative day one due to withdrawal of care by family. Surgical pathology showed a mucinous cystadenoma of the right ovary, chronic cervicitis, and atrophic endometrium with focal abscess. There was no noted malignancy or dysplasia. Figure 4 shows the purulent-appearing intrauterine cavity.

## Discussion

Any gross disruption of the cervical canal may inhibit the ability of the uterus to properly clear its secretions. This leads to uterine infections and may culminate in frank perforation. Although more common in post-menopausal women, the spontaneous rupture of the uterus in the setting of pyometra is still very rare [4,5]. Ruptured pyometra has a median age of 73.8 years of age and carries a 25-40% mortality rate following rupture [1,6]. As an intraperitoneal structure, when the uterus ruptures due to an underlying infection, spillage of pus into the abdominal cavity may lead to a surgical emergency. Some etiologies to consider for causing cervical stenosis and secondary pyometra include benign and malignant neoplasms, post-radiation cervicitis, atrophic cervicitis, senile cervicitis, gross congenital anatomic abnormalities, age-related endometritis, infective endometritis, and retained intrauterine device (IUD) or foreign body [5]. Pyometra secondary to malignancy carries a much more grave prognosis and should be the top differential diagnosis until proven otherwise [7]. Comorbidities that have been associated with increased association of rupture include type 2 diabetes mellitus, underlying fecal or urinary incontinence, immobility, obesity, malnutrition, poor hygiene, immunocompromised, increased sexual activity, genital atrophy, and cervical insufficiency [1]. The most common organisms in pyometra are flora of the genitourinary tract such as *Escherichia coli*, *Bacteroides*, *Streptococcus*, and other anaerobes [1,5]. In this case, our patient’s purulent abdominal fluid cultures grew *Bacteroides fragilis* and *Escherichia coli,* which is consistent with expected organisms from perforated pyometra.

Presentation of a ruptured uterus may be nonspecific such as in our case, with patients presenting only with nonspecific abdominal findings. Typical presentation includes purulent vaginal discharge, postmenopausal bleeding, and generalized abdominal pain [8]. Other nonspecific symptoms may include nausea, vomiting, diarrhea, fevers, and generalized weakness as was present in our patient [1]. Complete blood count may be significant for low hemoglobin and leukocytosis, although low sensitivity. Abdominal imaging in the setting of rupture will show pneumoperitoneum. Other nonspecific abdominal imaging findings may include periuterine edema, pneumouterus, abnormal air-fluid levels within the uterus, and gas within the endometrial canal [3,9]. The mainstay of treatment for spontaneous rupture of the uterus in the setting of pyometra is total hysterectomy with bilateral salpingo-oophorectomy, broad-spectrum antibiotics, abdominal washout, and postoperative ICU admission for close monitoring [1].

## Conclusions

Though correct preoperative diagnosis is always optimal, in the setting of pneumoperitoneum with hemodynamic instability, the definitive treatment is emergent exploratory laparotomy. In the presence of uterine pyometra, intraoperative gynecologic consultation should be obtained. Timely treatment with gynecologic surgery, early administration of broad spectrum antibiotics, abdominal washout, and ICU admission remain the mainstay of treatment. Keeping a broad differential but having a high level of suspicion while closely reviewing abdominal imaging makes the diagnosis more attainable. Clinicians should be aware that, although rare, perforated pyometra is a potential source for pneumoperitoneum and purulent peritonitis.
